# Integrated mRNA and microRNA transcriptome analysis reveals miRNA regulation in response to PVA in potato

**DOI:** 10.1038/s41598-017-17059-w

**Published:** 2017-12-05

**Authors:** Yanlin Li, Xinxi Hu, Jiren Chen, Wanxing Wang, Xingyao Xiong, Changzheng He

**Affiliations:** 1 College of Horticulture and Landscape, Hunan Agricultural University, Changsha, 410128 China; 2 Hunan Provincial Key Laboratory of Crop Germplasm Innovation and Utilization, Changsha, 410128 China; 3 Hunan Provincial Engineering Research Center for Potatoes, Changsha, Hunan 410128 China; 4 The Institute of Vegetables and Flowers, Chinese Academy of Agriculture Sciences, Beijing, 100081 China

## Abstract

Potato (*Solanum tuberosum* L.) is the fourth most important crop worldwide. Potato virus A (PVA) is one of the most harmful viruses infecting potatoes. However, the molecular mechanisms governing the responses to PVA infection in potato at the transcriptional and post-transcriptional levels are not well understood. In this study, we performed both mRNA and small RNA sequencing in potato leaves to identify the genes and miRNAs involved in the response to PVA infection. A total of 2,062 differentially expressed genes (DEGs) and 201 miRNAs (DEMs) were identified, respectively. Gene ontology (GO) and KEGG analysis revealed that these DEGs were involved in the transduction of pathogen signals, transcriptional reprogramming, induction of hormone signaling, activation of pathogenesis-related (PR) genes, and changes in secondary metabolism. Small RNA sequencing revealed 58 miRNA-mRNA interactions related to PVA infection. Some of the miRNAs (stu-miR482d-3p, stu-miR397-5p, etc) which target PR genes showed negative correlations between the DEMs and DEGs. Eight of the DEGs and three DEMs with their target genes were further validated by quantitative real time-PCR (qRT-PCR). Overall, this study provides a transcriptome-wide insight into the molecular basis of resistance to PVA infection in potato leaves and potenital candidate genes for improving resistance cultivars.

## Introduction

Potato (*Solanum tuberosum* L.) is the fourth most important crop wordwide for human consumption and industrial food processing. Due to the high nutritional value and the simplicity of its propagation by vegetative reproduction, the potato has become increasingly popular. However, cultivated potatoes, like many other plants, are exposed to diverse abiotic and biotic stresses, especially the host of a broad range of pathogens^1^. As a vegetatively propagated crop, potato is prone to virus infection during propagation, and this is one of the most challenging problems affecting potato production^2^. In China, six viruses, potato virus Y (PVY), potato virus X (PVX), potato virus A (PVA), potato virus S (PVS), potato leafroll virus (PLRV) and potato virus M (PVM), occur with a high incidence^3,4^. As the world’s largest potato-producing country, virus infection is one of the most challenging problems affecting potato production in China^4^.

PVA (*Potyvirus* genus, Potyviridae family) is one of the most harmful viruses infecting potatoes, reaching 40% of yield losses^2^. PVA can cause various symptoms, ranging from mild mosaic to severe leaf necrosis, depending on the potato cultivar and virus strain^5^. When the potato infected with mixed PVX and PVA, more-severe foliar symptoms and greater yield losses will occur^2^. Studies of the mechanisms of resistance to PVA in diploid potatoes have revealed that recessive and dominant genes interfere with the vascular transport of PVA^6^. Recently, a Chinese isolate of PVA was characterized and a genome recombination was found at the 3′-proximal end of the genome^2^. The results of the study supported the hypothesis that genome recombination had occurred in the evolution of PVA variants.

Global gene expression profiling of potato responses to abiotic and biotic stress have been studied previously^7–9^. And the availability of the potato genome sequences and next generation sequencing approaches, such as RNA sequencing (RNA-seq), have benefitted potato researchers. Recently, using RNA-seq, transcriptome responses to *Phytophthora infestans* inoculation in potato tubers have suggested that the hypersensitive response was likely to be a general form of resistance against late blight even in potato tuber^7^. RNA-seq profiling of a tolerant potato cultivar revealed that a quantitative defense response against *Pectobacterium carotovorum* ssp brasiliense was employed^1^. Quantitative proteomics and transcriptomics of potato in response to *P*. *infestans* identified the hypersensitive and effector targets^10^. A comparison of the transcriptional changes in the leaves of potato upon inoculation with PVY showed that the incompatible and compatible reactions in the resistant variety ‘Premier Russet’ shared more similarities, in particular during the initial response than the susceptible variety^11^. Although interaction transcriptome dynamics between potato and various pathogens have been reported, to our knowledge, no transcriptome studies have been focused specifically upon how potato responds to PVA.

Non-coding RNAs, including microRNAs (miRNAs) involved in plant biotic and abiotic stress to regulate gene expression at the post-transcriptional level^12,13^. In rice, viral-inducible miR319 suppressed JA-mediated defense to facilitate virus infection and symptom development^14^. When viral infection, osa-miR528 was preferentially associated with AGO18 and was elevated L-asorbate oxidase activity, enhancing the antiviral defense in rice^15^. Virus infections can also affect the accumulation of various miRNAs in potato. Overexpression of stu-miR482e down-regulated the target gene NBS-LRR protein, and enhanced sensitivity to *Verticillium dahliae* infection in potato^16^. Using a computational approach, 36 miRNAs were found to potentially target the PVY genome at 101 loci^17^. The co-infection of *Nicotiana benthamiana* with PVX or PVY altered the accumulation of miR156, miR171, miR168 and miR398 and their target transcripts^18^. These studies have indicated that miRNAs play important roles in the regulation of virus infection.

To obtain a global molecular understanding of one of the world’s most important crop diseases, we conducted an integrated mRNA and miRNA transcriptome analysis between PVA and potato. We further detected inversely correlated expression changes in the target transcripts of these miRNAs. These results provide insights into the interactions that occur on transcriptional and post-transcriptional regulation during the PVA infection.

## Materials and Methods

### Plant materials

The plantlets of potato ‘A6’ (*Solanum demissum* × Aquila) which is a PVA hypersensitive variety were cultivated in the protected field at Hunan Agricultural University (Changsha, Huann, China). After 40 days, the plantlets were cultured in MS medium and transferred to an incubator at 25 °C under 16 h light/8 h dark conditions. Potato leaves were collected at time point 0 h (pre-inoculation, CK) and at 24 and 60 hours post inoculation (hpi) (24 hpi, PVA_24 h and 60 hpi, PVA_60 h). And the leaves without PVA inoculation at 24 h and 60 h (WT_24 h and WT_60 h) were also harvested as a control, respectively. Mosaic leaves can be observed when the potato leaves infect PVA. Potato leaves from at least three individual plants were collected for each sample with three biological replicates.

### RNA extraction, library construction and sequencing

Total RNA was extracted from the potato leaves using an RNeasy Plant Mini Kit (Qiagen, Germany) according to the manufacturer’s protocol. The extracted RNA was treated with Dnase I (Promega, USA) to remove the contaminated DNA and detected by 1.0% agarose gel electrophoresis. The RNA concentration and integrity were measured with a Nanodrop2000 and Bioanalyzer 2100 system (Agilent Technologies, CA, USA).

For RNA-seq, 3 μg of total RNA from each sample was used for library preparation using a TruSeq Stranded Total RNA Sample Preparation kit (Illumina, San Diego, USA). RNA was fragmented into small pieces and then first-strand cDNA was synthesized with SuperScript II reverse transcription (Invitrogen, CA, USA). After purification, the second-strand cDNA library was synthesized, following several rounds of PCR amplification. For small RNA sequencing, 5 μg of total RNA was ligated to a 5′ RNA adaptor and 3′ RNA adaptor according to the manual of NEBNext^®^ Multiplex Small RNA Library Prep Set for Illumina (NEB, MA,USA). The RNAs were reverse transcribed to cDNAs, following PCR amplification. Subsequently, the libraries were purified and sequenced by Biomarker Technologies (Beijing, China) on an Illumina Hiseq2500 platform with 125 bp paired-end and 50 bp single-end, respectively. Three biological replicates were performed for each sample. All the clean reads were deposited in the National Center for Biotechnology Information (NCBI) Sequence Read Archive under the accessions: SRP098975 (RNA-seq) and SRP098770 (small RNA sequencing).

### Analysis of differentially expressed genes (DEGs) and annotation

Raw reads were quality checked with the FastQC package (http://www.bioinformatics.babraham.ac.uk/projects/fastqc/), and adaptor sequences and low quality reads were removed. We used relatively stringent criteria for quality control by removing the reads containing Ns and the reads whose PHRED-like score was less than 30. The clean reads were mapped to the reference genome of potato using Tophat 2 (version 2.0.13)^19,20^. Transcript reconstruction was conducted by Cufflinks software (version 2.2.1)^21^. DESeq was used to make read counts and to identify DEGs. We used the fragments per kilobases of exon per million fragments mapped (FPKM) values to examine the expression level. FPKM = C/LN. Where C is the number of mapped reads on the transcript, L is the length of transcript (kb), N is the total number of mapped reads (Millions). Using Fisher’s exact test, a False Discovery Rate (FDR) was determined with a threshold of 0.05 and |log2(fold change)| ≥ 1) to recognize the significance of the differences in gene expression.

### Identification of miRNA and target prediction

After trimming adaptor sequences and low quality reads, the clean reads were aligned to the reference genome using SOAP software, with a default parameter. The sRNA reads with no more than two mismatches to the non-coding RNA in the Rfam database, including tRNA, rRNA, snRNA and snoRNA, were excluded. The conserved miRNAs were identified by comparing the sRNA reads with the known plant miRNAs in the miRBase 20.0 (http://www.mirbase.org)^22^. The unannotated reads were used for prediction of novel miRNAs using miRDeep2^23^ and Mfold^24^. The miRNA target genes were predicted using psRNATarget with default parameters^25^.

Differentially expressed miRNAs (DEMs) between samples were performed using the DEGseq R package. The FDR < 0.01 and log2|fold change| > 1 was set as the threshold for significantly different expression.

### Gene ontology and KEGG pathway enrichment analysis

The DEGs and the targets of DEMs were annotated with gene ontology (GO) terms to investigate putative functions. A GO enrichment analysis was performed using the TopGO R package and AgriGO program^26^. The statistical significance of GO terms were measured by a Fisher’s exact test corrected by FDR of <0.05. A Kyoto Encyclopedia of Genes and Genomes (KEGG) (http://www.genome.jp/kegg/)^27^ pathway were conducted using KOBAS3.0 (http://kobas.cbi.pku.edu.cn/) with an enrichment *p-*value < 0.05.

### Quantitative real-time PCR (qRT-PCR) Analysis

Total RNA was extracted from the samples using Trizol (Takara, Dalian, China) and treated with RNase-free Dnase I (Promega, USA). Approximately 2 μg of total RNA was reverse transcribed using a Fermentas RevertAid First Strand cDNA Synthesis Kit (Fermentas, USA). The qRT-PCRs analysis of the miRNA and mRNA were performed using the Premix Ex Taq^TM^ kit (Takara, Japan) on the StepOne plus PCR platform (Applied Biosystems). For miRNA, the expression level was detected by stem-loop RT-PCR using miRNA-specific stem-loop primers^28^. The reactions were incubated for 30 min at 16 °C, followed by pulsed RT of 60 cycles at 30 °C for 30 s, 42 °C for 30 s and 50 °C for 1 s and finally the reactions were terminated at 70 °C for 5 min. The qRT-PCR reactions were conducted with the following protocol: 95 °C for 10 min, followed by 40 cycles of 95 °C for 15 s and 56 °C for 30 s and 72 °C for 15 s. *EF1α* was used as the internal control for mRNAs and miRNAs^29^. All the primers used in this study were listed in Table S1. A melting curve analysis was performed to determine the specificity of the products. The qRT-PCR reactions were performed with three biological replicates and the relative gene expression level was analyzed using the comparative 2^ΔΔ^CT method^30^.

## Results

### Overview of transcriptome dynamics and small RNA sequencing

**Figure 1:**
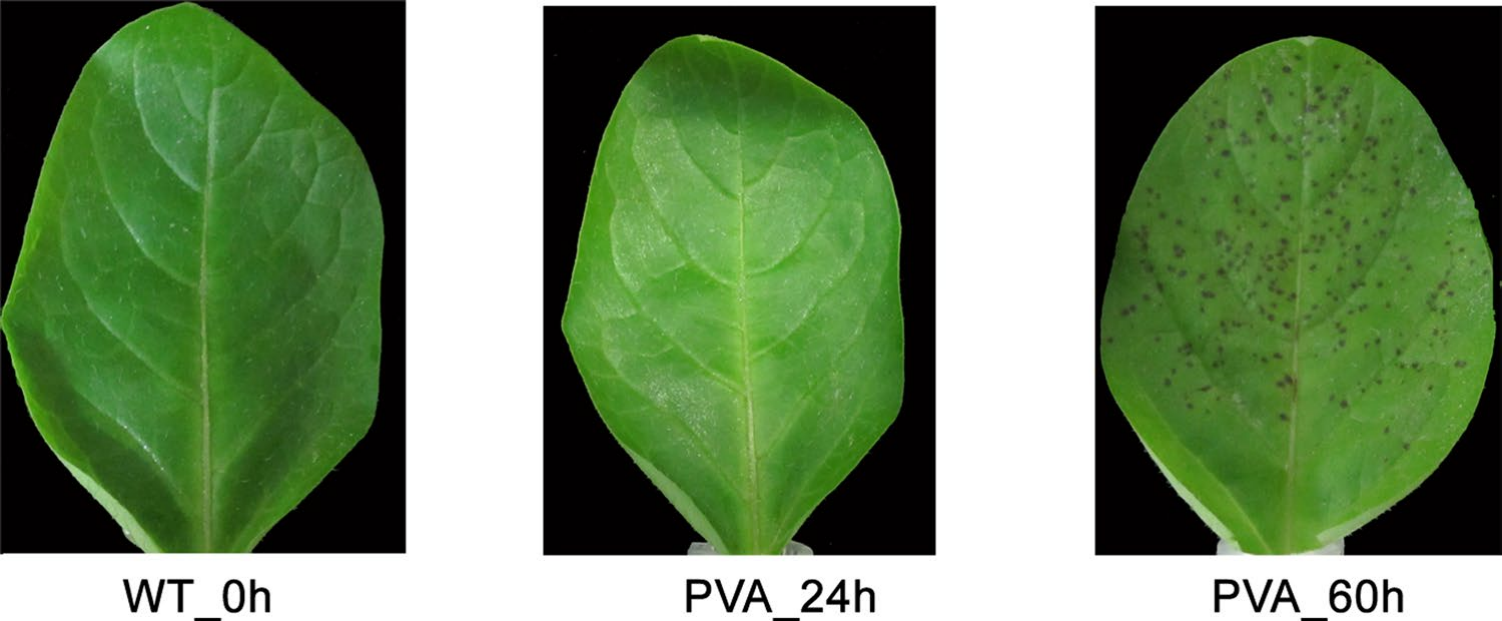
The morphologies of potato leaves with and without (WT) PVA infection at different time points (0 h, 24 h and 60 h).

PVA can cause varying degrees of symptoms, ranging from mild mosaic to severe leaf necrosis. In this study, we can observe mottle, mosaic potato leaves at 60 hpi (Fig. 1). To obtain a comprehensive transcriptome profile of potato in response to PVA, 15 libraries of leaves were constructed with or without inoculation from different time points (0 h, 24 h and 60 h). Totally, 97.96 Gb clean reads was obtained with an average of 6.53 Gb for each sample (Table S2). For each time-point sample, the three biological replicates showed a high correlation with each other (Figure S1A).

Furthermore, the corresponding small RNA libraries at the three timpe points were also constructed for deep sequencing. After removing adaptors, the low-quality reads, including reads with lengths < 18 nt or >30 nt, the remaining clean reads were ranging from 12,168,812 clean reads (PVA_60h-rep1) to 19,336,916 clean reads (0h-rep1) with an average of 223,337,918 reads (Table S2). The three biological replicates were strongly correlated among samples (Figure S1B). About 50% of the total sRNAs were common to two different libraries, while only a relatively small fraction (~12%) of the unique sequence reads were shared between adjacent libraries (Figure S2).

### DEGs of potato leaves in response to PVA infection

**Figure 2:**
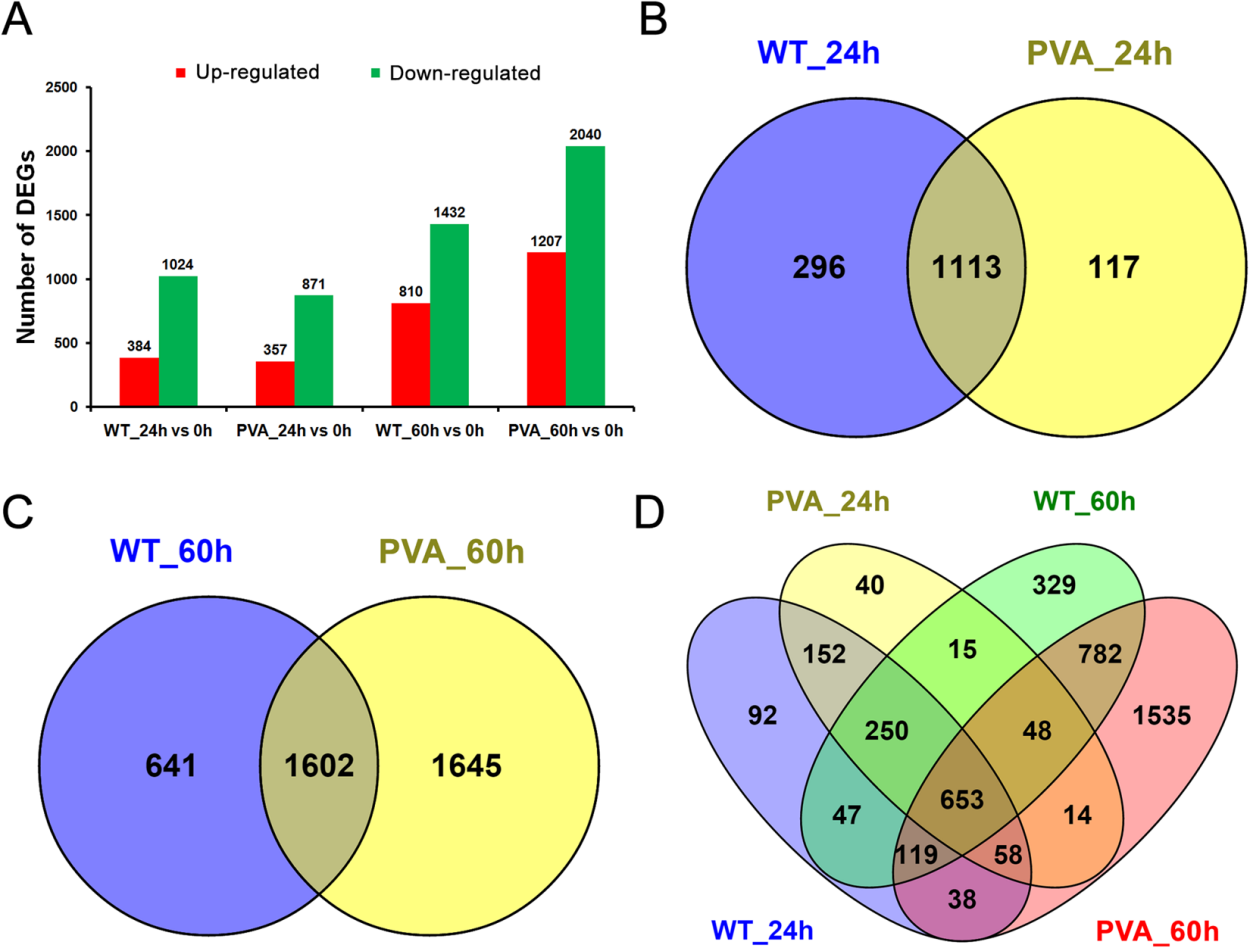
Comparative analysis of differentially expressed genes (DEGs) at 24 and 60 hpi. (**A**) Numbers of DEGs compared between two samples (WT_24 h vs 0 h, PVA_24 h vs 0 h, WT_60 h vs 0 h, and PVA_60 h vs 0 h). (**B**) Venn diagram showing the overlap of DEGs between WT_24 h and PVA_24hpi. (**C**) Venn diagram showing the overlap of DEGs between WT_60 h and PVA_60 hpi. (**D**) Venn diagram showing the overlap of DEGs among the four samples compared with no inoculation (0 h).

To investigate the expression profiles of genes in potato leaves in response to PVA infection, expression levels were compared based on the FPKM values. Using DEseq software, the gene expression between the samples and WT line (without inoculation at 0 h) were compared. We identified 1,408, 1,228, 2,242 and 3,247 DEGs among the time points (24 h and 60 h), respectively (Fig. 2A). Totally, 2,062 DEGs were identified in all samples by removing the repeats and most of these DEGs were down-regulated at 60 hpi (Fig. 2A and Table S3). Overlapping the DEGs between the samples with and without inoculation showed that 117 and 1,645 DEGs were specific expressed at 24 h (PVA_24 h) and 60 h (PVA_60 h) of inoculation, respectively (Fig. 2B,C). Among the four samples, a total of 653 DEGs were present over the time course (24 h and 60 h) (Fig. 1D). These DEGs included ethylene response factors, chitinase, psbP, peroxidase, and so on (Table 1). Two genes associated with disease resistance, PGSC0003DMG400008673 (chitinase) and PGSC0003DMG400005109 (PR) were highly expressed at PVA_60 h (Table 1).

**Table 1:** The differential expression of some PVA-responsive genes in potato leaves.

Gene ID	Annotation	WT_0 h	WT_24 h	PVA_24 h	WT_60 h	PVA_60 h
**Pathogen-related genes**						
PGSC0003DMG400005109	pathogenesis-related protein	0.05	5.38	5.65	8.24	47.72**
PGSC0003DMG402016981	TMV resistance protein	3.03	0.35	0.35	0.37	0.49
PGSC0003DMG400008673	chitinase	1.00	12.07	4.25**	9.56	50.12**
PGSC0003DMG400032771	bidirectional sugar transporter	2.35	154.83	15.14**	95.56	24.35**
PGSC0003DMG400008517	bidirectional sugar transporter	1.86	139.68	13.23**	104.57	24.21**
PGSC0003DMG400002901	chlorophyll a-b binding protein	204.65	22.81	35.07	2.84	0.60**
PGSC0003DMG400016504	photosystem I reaction center subunit VI-2	720.57	123.20	151.52	93.59	23.68**
PGSC0003DMG400020505	photosystem I reaction center subunit psaK	559.59	53.04	89.23	44.35	10.19
PGSC0003DMG400002312	photosystem II 10 kDa polypeptide	0.30	10.57	9.20	61.75	5.55
PGSC0003DMG400024531	psbP-like protein 1	206.13	40.48	47.38	8.14	3.23**
PGSC0003DMG400030834	thylakoid lumenal 29 kDa protein	176.72	36.29	42.59	7.19	3.13
PGSC0003DMG400027614	cell wall peroxidase	27.81	0.70	0.43	1.22	3.16
PGSC0003DMG400014867	peroxidase	14.21	117.96	92.59	69.20	191.89
PGSC0003DMG400011640	peroxidase 4	3.03	64.59	46.17	110.65	281.83
PGSC0003DMG400030919	blue copper protein	0.13	3.29	3.91	20.96	57.19*
PGSC0003DMG401009045	DELLA protein GAI1	1.47	4.89	7.23	4.52	8.22
PGSC0003DMG400026461	DREB1	20.02	2.50	1.31	4.39	1.08**
PGSC0003DMG400007994	tuber-specific and sucrose-responsive element	26.63	0.92	1.66	1.01	1.84
PGSC0003DMG400021422	BTB/POZ and TAZ domain-containing protein	1.15	5.03	4.58	5.32	20.51**
PGSC0003DMG400012017	pectin methylesterase inhibitor	54.58	3.67	6.79	0.19	0.45
PGSC0003DMG400018328	proteinase inhibitor	43.59	557.44	283.07	549.98	2447.76**
PGSC0003DMG400025472	MAP kinase kinase kinase	7.84	0.02	0.05	0.01	0.18
PGSC0003DMG400008296	LRR receptor-like serine/threonine-protein kinase	20.53	1.82	3.03	1.48	2.07
PGSC0003DMG400024795	LRR receptor-like serine/threonine-protein kinase	6.05	0.91	1.14	0.50	0.07
PGSC0003DMG400028259	growth-regulating factor 2	1.26	0.07	0.06	0.11	0.04
PGSC0003DMG400010422	pentatricopeptide repeat-containing protein	37.97	6.70	8.11	0.14	0.35
PGSC0003DMG400027963	Ga20 oxidase	5.80	0.60	0.75	0.37	0.07
PGSC0003DMG401025908	salicylic acid-binding protein 2	8.29	0.53	0.39	0.37	0.13
**Transcription factors**						
PGSC0003DMG400007788	WRKY transcription factor 28	0.23	1.80	1.44	3.11	7.19
PGSC0003DMG400011603	auxin-binding protein ABP19a	128.46	3.93	7.30	2.18	0.36
PGSC0003DMG400001604	auxin-induced protein 15 A	7.38	0.48	0.21	0.83	1.37
PGSC0003DMG400015607	IAA-amino acid hydrolase ILR1	64.56	5.86	6.42	3.04	7.59
PGSC0003DMG400015531	zinc finger protein ZAT11	51.51	0.51	0.73	1.36	10.21**
PGSC0003DMG400000066	ethylene-responsive late embryogenesis	240.06	22.23	10.74	3.57	2.25
PGSC0003DMG400002899	ethylene-responsive transcription factor	45.91	0.54	0.95	2.97	0.51
PGSC0003DMG400000811	AP2/ERF	131.20	26.24	28.39	18.81	31.34
PGSC0003DMG400001338	NAC	0.81	7.17	5.99	15.64	6.54
PGSC0003DMG400033047	NAC	2.78	26.34	22.06	20.60	33.02
PGSC0003DMG400004808	small heat shock protein	8.07	64.67	67.14	246.13	128.75
PGSC0003DMG400027283	heat stress transcription factor B-3	0.81	6.57	4.98	9.65	61.47
PGSC0003DMG400000444	heat shock cognate 70 kDa protein	0.78	13.68	14.61	144.25	46.73*
PGSC0003DMG400011977	small heat shock protein	0.43	7.97	11.61	106.19	38.43*
PGSC0003DMG400008187	class II small heat shock protein Le-HSP17.6	1.27	10.69	8.92	156.25	22.29**

Significant differences in the expression level between CK and PVA at 24 h or 60 h were evaluated using Student’s *t* test (*P < 0.05, **P < 0.01).

### Transcriptional changes in response to PVA inoculation

**Figure 3:**
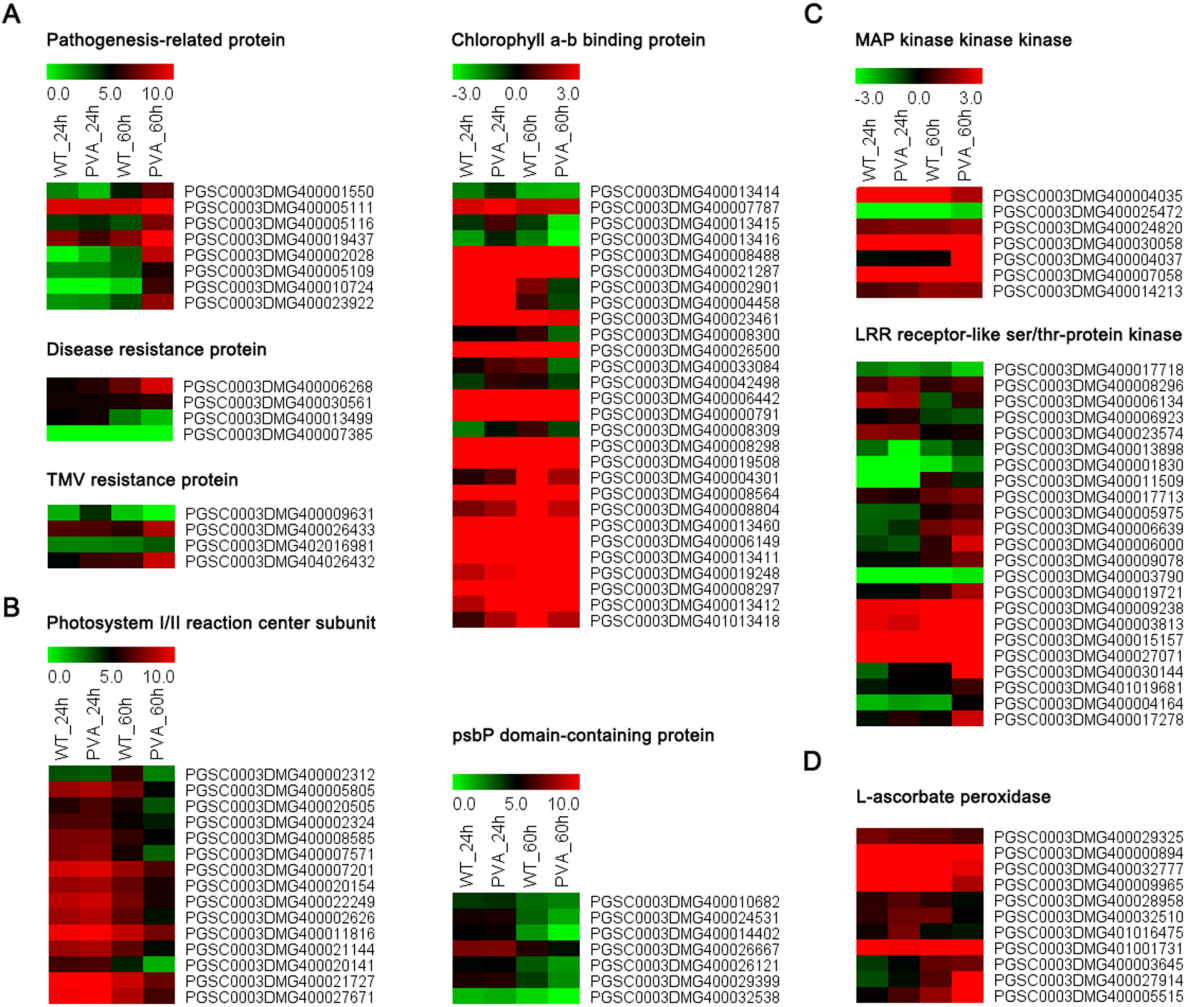
Heatmaps of the differentially expressed genes (DEGs) at different time points (24 and 60 hpi). (**A**) Pathogenesis-related protein, disease resistance protein and TMV resistance protein. (**B**) Photosystem I/II reaction center subuit, chlorophyll a-b binding protein and psbP. (**C**) MAP kinase kinase kinase and LRR receptor-like ser/thr-protein kinase. (**D**) L-ascrorbate peroxidase.

Among the identified DEGs, many genes involved in disease resistance and photosystem were differentially expressed (Fig. 3A,B, Tables 2, and S3). Of them, 8, 4 and 5 DEGs were belonged to pathogenesis-related (PR) protein, disease resistance and TMV resistance protein, respectively (Table S4). Most of these DEGs were up-regulated at 24 hpi or 60 hpi, indicating that they played a role in disease resistance (Fig. 3A). Other resistance genes (for example, PGSC0003DMG400005656, NB-LRR protein) were also up-regulated at 24 hpi (Table S3). Moreover, a total of 56 photosynthesis-related genes were differentially expressed after PVA inoculation, including photosystem I/II reaction center subunit, psbP, chlorophyllase and chlorophyll a/b binding protein (Fig. 3B and Table S4). Of these genes, 28 and 15 DEGs, which encode chlorophyll a-b binding protein and photosystem I/II reaction center subunit, respectively, were down-regulated at 60 hpi (Fig. 3B). In addition, many of the kinases, including MAP kinase kinase kinase and LRR receptor-kile ser/thr protein kinase, were up-regulated at 60hpi (Fig. 3C and Table S4). Eleven genes encoding L-ascorbate oxidase (AO) were also differentially expressed and their expression levels increased over the times (Fig. 3D and Table S4).

**Table 2:** The differential expression of disease resistance annotated-genes in responsive to PVA in potato leaves.

Gene ID	Annotation	WT_0 h	WT_24 h	PVA_24 h	WT_60 h	PVA_60 h
**Pathogen-related genes**						
PGSC0003DMG400001550	pathogenesis-related protein	0.062	5.202	2.379	22.666	123.619**
PGSC0003DMG400002028	pathogenesis-related protein	1.484	1.104	2.504	9.063	427.894**
PGSC0003DMG400005109	pathogenesis-related protein	0.047	5.378	5.653	8.237	47.720
PGSC0003DMG400005111	PR1	138.868	633.769	576.092	606.129	4487.84**
PGSC0003DMG400005116	PR1	2.668	14.066	18.278	10.712	182.748
PGSC0003DMG400010724	pathogenesis-related protein	8.767	0.722	1.022	1.835	71.086
PGSC0003DMG400019437	pathogenesis-related protein	35.047	194.857	79.484	210.685	5651.992**
PGSC0003DMG400023922	pathogenesis-related protein	0.347	4.208	4.776	11.634	241.268
**Disease resistance protein**						
PGSC0003DMG400006268	disease resistance protein SlVe2	7.823	1.120	1.364	2.261	5.841
PGSC0003DMG400013499	Pto disease resistance protein	1.703	1.030	1.158	0.365	0.219
PGSC0003DMG400030561	Disease resistance family protein	4.894	1.179	1.152	1.260	1.451
PGSC0003DMG400007385	CC-NB-LRR protein	0.735	0.037	0.068	0.096	0.025
**TMV resistance protein**						
PGSC0003DMG400009631	TMV resistance protein	2.186	0.237	0.697	0.203	0.107
PGSC0003DMG400026433	TMV resistance protein	8.988	1.955	1.852	1.495	4.108*
PGSC0003DMG402016981	TMV resistance protein	3.029	0.349	0.354	0.374	0.489
PGSC0003DMG404026432	TMV resistance protein	13.903	0.964	1.616	1.675	5.095*

Significant differences in the expression level between CK and PVA at 24 h or 60 h were evaluated using Student’s *t* test (*P < 0.05, **P < 0.01).

### Expression of transcription factors (TFs) and phytohormone related genes

**Figure 4:**
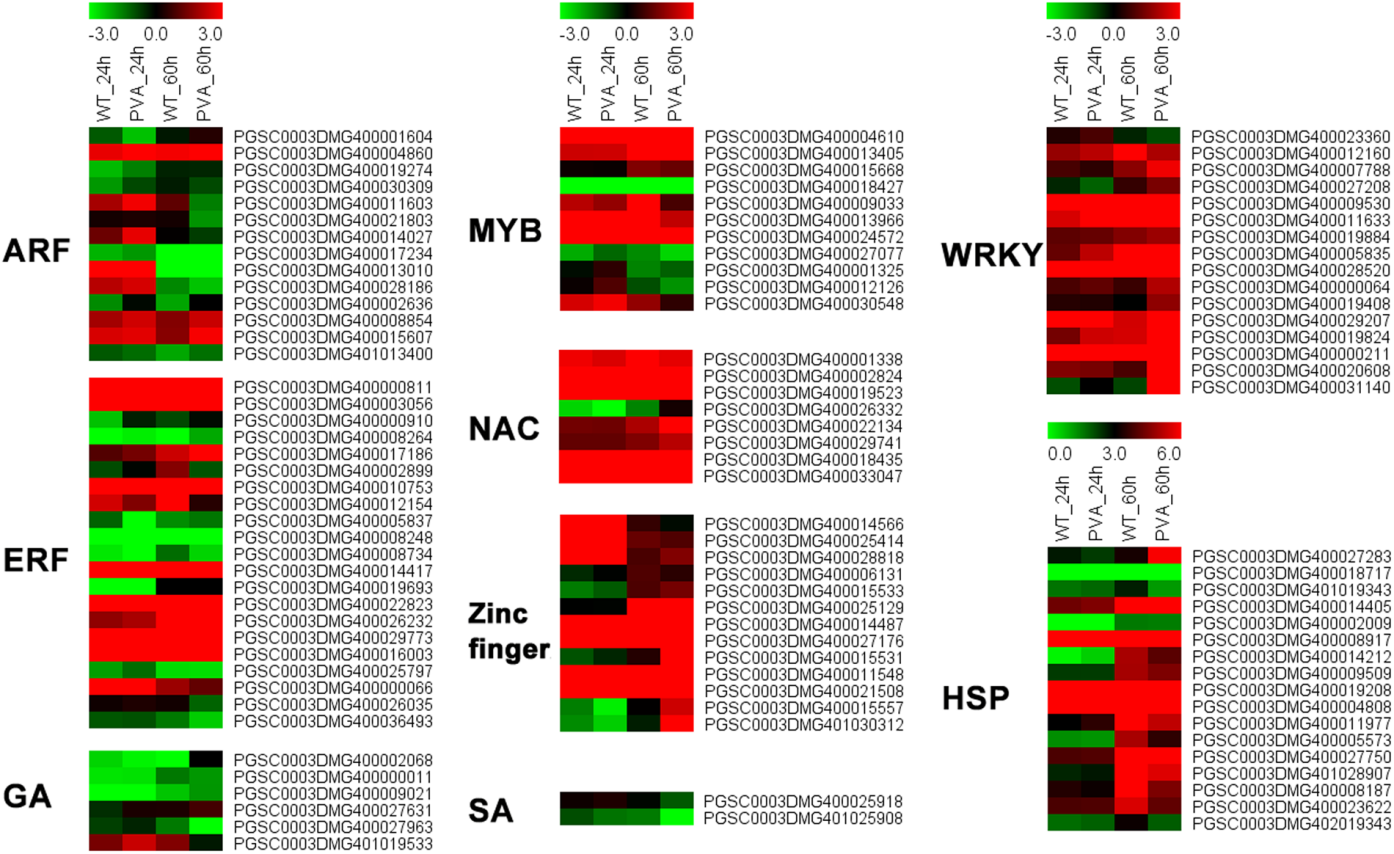
Heatmaps of the differentially expressed genes (DEGs) and Transcription factors at different time points (24 and 60 hpi).

Transcription factors (TFs) play important roles in response to abiotic stress in plants^31^. In the present study, we found that 103 TFs, including HSP, ERF, ARF, AP2, MYB, Zine finger, WRKY, and NAC, were differentially expressed following PVA infection (Fig. 4, Tables 1 and S4). The ERF family with 24 DEGs was the largest TF family responding to PVA infection (Table S4). And a total of 15 DEGs belonging to the WRKY or HSP family were identified, respectively (Fig. 4). Most of the differentially expressed WRKYs and HSPs were up-regulated, while ARFs and zinc finger proteins were typically down-regulated (Fig. 4). These TFs had different expression patterns in response to PVA infection, suggesting a variety of regulatory mechanisms.

A total of 43 DEGs involved in signal transduction pathways were identified, including ethylene, auxin (IAA), jasmonic acid (JA), salicylic acid (SA) and abscisic acid (ABA) pathways (Fig. 4 and Table S4). We identified 6 and 3 DEGs that participated in gibberellin (GA) and IAA pathway, respectively (Table S4). And most of these DEGs were up-regulated at 24 hpi, while they were down-regulated at 60 hpi (Fig. 4 and Table S4). One of these DEGs, PGSC0003DMG400002930 (jasmonate ZIM-domain protein 1), which is involved in the JA pathway showed a significant induction after PVA infection, especially at 60hpi (Table S3). Two genes PGSC0003DMG400025918 and PGSC0003DMG401025908, encoding salicylic acid (SA)-binding protein, were also differentilly expressed during PVA infection (Fig. 4 and Table S4).

### Functional classification of DEGs

**Figure 5:**
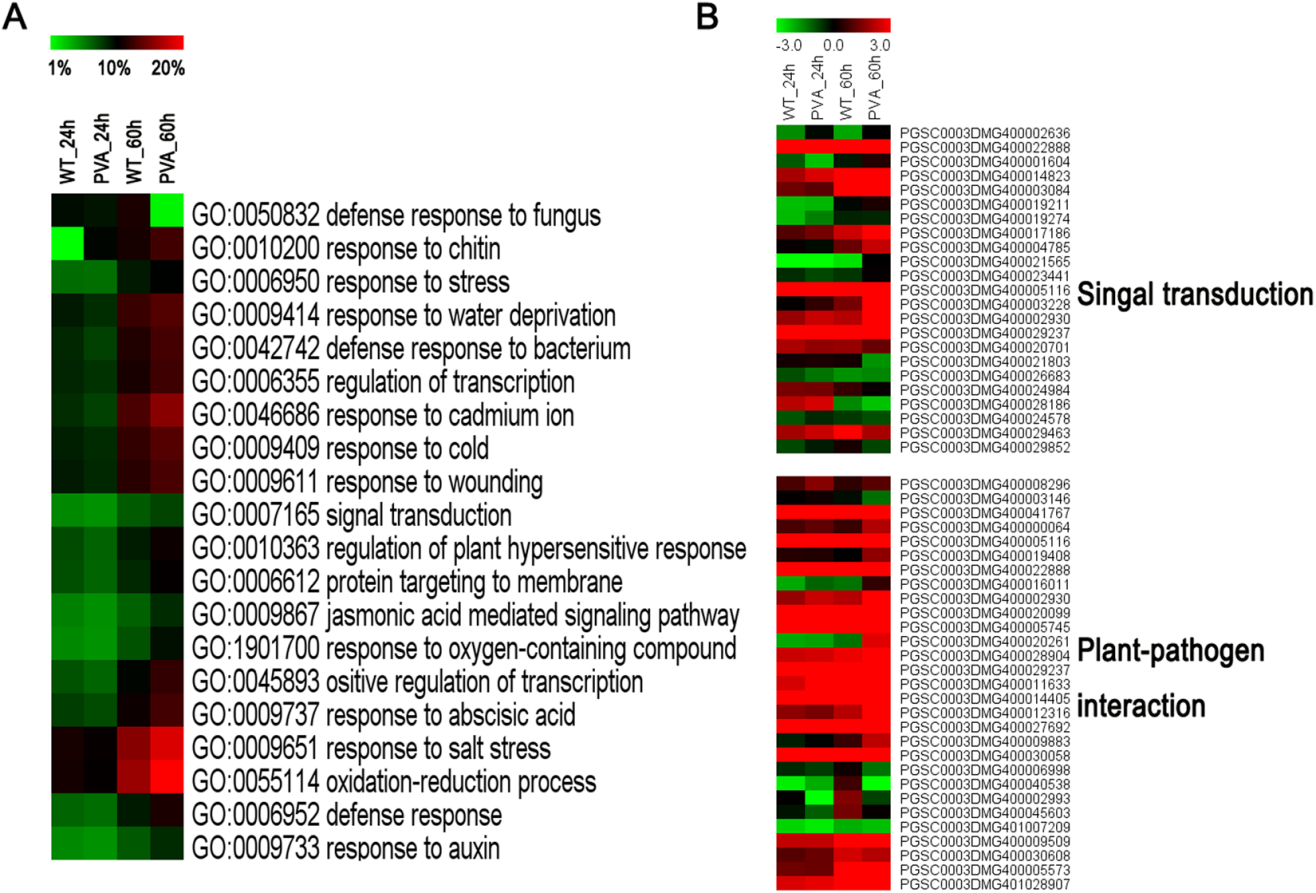
Gene orthology (GO) analysis of differentially expressed genes (DEGs) at different time points (24 and 60 hpi) and heatmaps of some DEGs. (**A**) TOP 20 GO enriched terms at 24 and 60 hpi. (**B**) Heatmaps of the enriched DEGs that are involved in ‘singal transduction’ and ‘plant-pathogen interaction’.

To infer the biological processes of DEGs in response to PVA, we conducted a GO analysis of the DEGs that were identifed in this study. Approximately 20 GO terms were enriched among all the three time points and treatments (Fig. 5A and Table 3). These most dominant terms included response to salt stress (GO: 0009651), oxidation-reduction process (GO: 0055114), response to chitin (GO: 0010200), defense response to fungus (GO: 0050832) and so on. Particularly, defense response to bacterium (GO: 0042742) and jasmonic acid mediated signaling pathway (GO: 0009867) were also enriched, with a total of 247 and 158 DEGs (removing the repeats), respectively (Table 3). The number of DEGs included in the dominant terms increased with the time of inoculation (Fig. 5A and Table 3).

**Table 3:** Top 20 differential enrichment of GO terms in response to PVA infection.

GO ID	Annotation	WT0hvsWT24h	WT0hvsPVA24h	WT0hvsWT60h	WT0hvsPVA60h	WT60hvsPVA60h
GO:0009651	response to salt stress	350 (10.94%)	331 (10.35%)	491 (15.35%)	596 (18.64%)	316 (9.88%)
GO:0055114	oxidation-reduction process	348 (10.88%)	327 (10.22%)	513 (16.04%)	639 (19.98%)	344 (10.76%)
GO:0010200	response to chitin	339 (10.60%)	313 (9.79%)	350 (10.94%)	402 (12.57%)	296 (9.26%)
GO:0050832	defense response to fungus	306 (9.57%)	296 (9.26%)	356 (11.13%)	422 (13.20%)	293 (9.16%)
GO:0009414	response to water deprivation	291 (9.10%)	269 (8.41%)	393 (12.29%)	426 (13.32%)	260 (8.13%)
GO:0009611	response to wounding	291 (9.10%)	274 (8.57%)	373 (11.66%)	413 (12.91%)	245 (7.66%)
GO:0009409	response to cold	284 (8.88%)	271 (8.47%)	386 (12.07%)	428 (13.38%)	249 (7.79%)
GO:0006355	regulation of transcription, DNA-templated	277 (8.66%)	263 (8.22%)	353 (11.04%)	399 (12.48%)	220 (6.88%)
GO:0042742	defense response to bacterium	275 (8.60%)	247 (7.72%)	363 (11.35%)	407 (12.73%)	276 (8.63%)
GO:0046686	response to cadmium ion	270 (8.44%)	247 (7.72%)	414 (12.95%)	494 (15.45%)	284 (8.88%)
GO:0009737	response to abscisic acid	250 (7.82%)	234 (7.32%)	338 (10.57%)	406 (12.70%)	227 (7.10%)
GO:0010363	regulation of plant-type hypersensitive response	235 (7.35%)	209 (6.54%)	288 (9.01%)	338 (10.57%)	260 (8.13%)
GO:0006612	protein targeting to membrane	231 (7.22%)	205 (6.41%)	274 (8.57%)	327 (10.23%)	255 (7.97%)
GO:0045893	positive regulation of transcription, DNA-templated	227 (7.10%)	205 (6.41%)	313 (9.79%)	384 (12.01%)	197 (6.16%)
GO:0006952	defense response	207 (6.47%)	196 (6.13%)	290 (9.07%)	355 (11.10%)	248 (7.75%)
GO:0006950	response to stress	198 (6.19%)	194 (6.07%)	291 (9.10%)	317 (9.91%)	183 (5.72%)
GO:0009867	jasmonic acid mediated signaling pathway	175 (5.47%)	158 (4.94%)	210 (6.57%)	271 (8.47%)	198 (6.19%)
GO:1901700	response to oxygen-containing compound	166 (5.19%)	154 (4.82%)	228 (7.13%)	302 (9.44%)	196 (6.13%)
GO:0007165	signal transduction	166 (5.19%)	156 (4.88%)	217 (6.79%)	242 (7.57%)	158 (4.94%)
GO:0009733	response to auxin	164 (5.13%)	155 (4.85%)	220 (6.88%)	272 (8.51%)	155 (4.85%)

**Figure 6:**
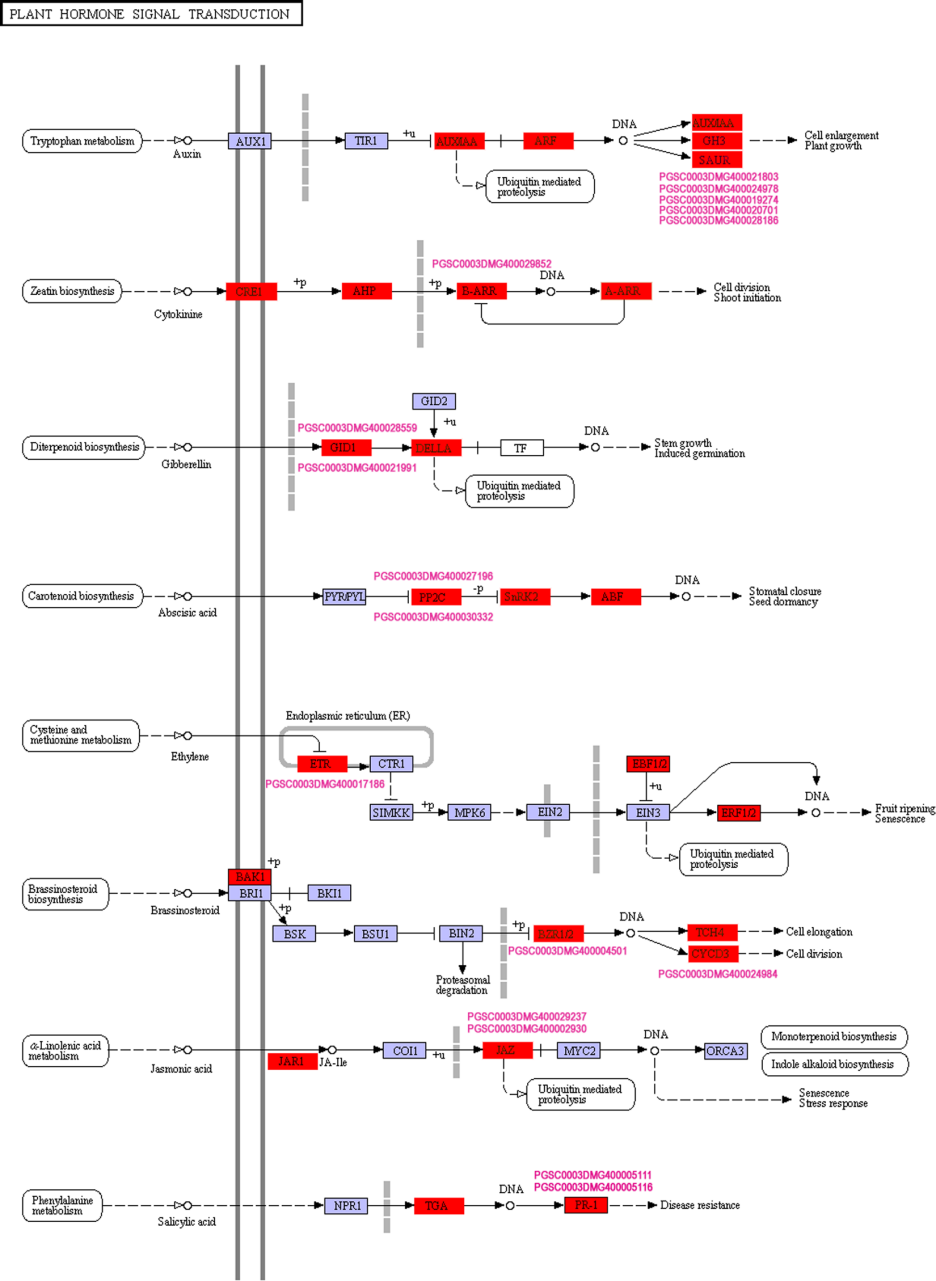
Schematic of the pathway category ‘Plant hormone signal transduction’. The map is from the Kyoto Encyclopedia of Genes and Genomes (KEGG) (http://www.genome.jp/kegg/)^27^.

To further investigate the biological functions of these DEGs, KEGG pathway analysis was also conducted. We identified 99 pathways that were significantly enriched in comparisons of inoculation PVA samples versus the control (Tables 4 and S5). It is worth noting that “Plant hormone signal transduction”, “Starch and sucrose metabolism” and “Plant-pathogen interaction” were significantly enriched (Table 4). In addition, some genes involved in “Photosynthesis” and “Brassinosteroid biosynthesis” were also differentially expressed (Fig. 3 and Table S5). Totally, there were 335 and 143 genes involved in “Plant hormone signal transduction” and “Plant-pathogen interaction”, respectively, including three phytohormones (ethylene, auxin, JA) and TFs (WRKYs and HSPs) (Fig. 5B and Table S5). Some of the DEGs involved in signal transduction pathway were showed in Fig. 6.

**Table 4:** Top 20 enriched KEGG pathways in response to PVA infection.

Pathway ID	Pathway	Genes in all (4515)
ko04075	Plant hormone signal transduction	335 (7.42%)
ko03010	Ribosome	262 (5.8%)
ko04141	Protein processing in endoplasmic reticulum	216 (4.78%)
ko03040	Spliceosome	191 (4.23%)
ko03013	RNA transport	176 (3.9%)
ko00190	Oxidative phosphorylation	172 (3.81%)
ko04120	Ubiquitin mediated proteolysis	159 (3.52%)
ko00230	Purine metabolism	157 (3.48%)
ko00500	Starch and sucrose metabolism	157 (3.26%)
ko00908	Zeatin biosynthesis	145 (3.21%)
ko04626	Plant-pathogen interaction	143 (3.17%)
ko00010	Glycolysis / Gluconeogenesis	135 (2.99%)
ko00240	Pyrimidine metabolism	125 (2.77%)
ko03008	Ribosome biogenesis in eukaryotes	119 (2.64%)
ko04144	Endocytosis	115 (2.55%)
ko00360	Phenylalanine metabolism	115 (2.55%)
ko00520	Amino sugar and nucleotide sugar metabolism	114 (2.52%)
ko03015	mRNA surveillance pathway	111 (2.46%)
ko00940	Phenylpropanoid biosynthesis	111 (2.46%)
ko03018	RNA degradation	109 (2.41%)

### Identification of miRNAs in potato leaves in response to PVA

After filtering the data set and blasting against the known mature miRNAs and miRNA precursors in miRBase 20.0 (http://www.mirbase.org), 190 conserved miRNAs were identified. In addition to the conserved miRNAs, the remaining sequences were aligned with the genome sequences of potato to identify novel miRNAs. Using miRDeep2 software, a total of 120 novel miRNAs were obtained from the five samples (Table S6). To confirm whether the sequences of these small RNAs were true miRNAs for potato leaves, the hairpin structures were determined by Mfold or RNAfold. As a result, all the precursors of these miRNAs possessed typical stem-loop structures (Table S6).

### Differential expression profiles of miRNAs associated with PVA infection

**Figure 7:**
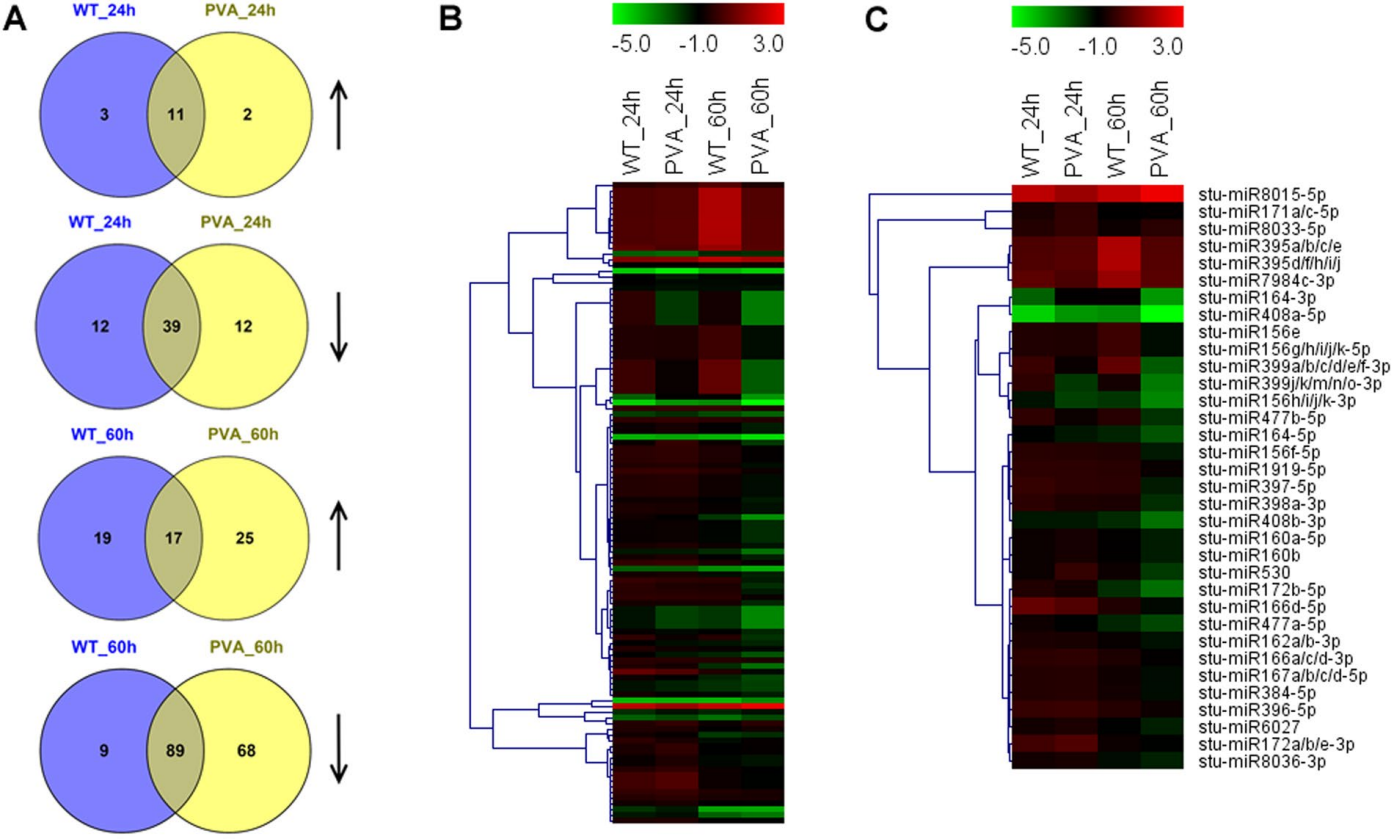
Overview and heatmaps of differentially expressed miRNAs (DEMs) at different time points (24 and 60 hpi). (**A**) Venn diagram showing the overlap of DEMs with up-regulation and down-regulation at 24 and 60 hpi, respectively. (**B**) Heatmap of the DEMs at 24 and 60hpi. (**C**) Heatmap of some conserved DEMs at 24 and 60hpi.

To compare the different miRNA expression profiles in response to PVA infection, a differential expression analysis of the miRNAs in potato leaves was performed between the four samples with 0 h (CK) of potato leaves, based on the normalized read count for each identified miRNA. A total of 113 conserved and 88 novel DEMs were found to be involved in PVA infection (Fig. 7 and Table S7). More DEMs were identified at 60 hpi than at 24 phi, having 25 and 68 DEMs up-regulated and down-regulated, respectively (Fig. 7A). The expression analysis showed that most of these DEMs decreased at 60hpi, including stu-miR164-5p, stu-miR397-5p and stu-miR408a-3p (Fig. 7B,C and Table S7). Moreover, a total of 585 target genes were identified for the 113 conserved miRNAs and 120 novel miRNAs (Table S8). As expected, most of the target genes for 113 miRNAs were conserved among other plants. For example, the target genes of stu-miR160a-5p and stu-miR172a-3p were ARF18 (PGSC0003DMG400017585) and AP2 (PGSC0003DMG400025390), respectively. Besides the miR482 family (stu-miR482a/b/d/e-3p and stu-miR482c), other miRNAs including stu-miR6024-3p, stu-miR6025, stu-miR6027 and stu-miR8038a-3p also targeted the disease resistance protein (Tables 5 and S8), indicating that these miRNAs may be involved in response to PVA.

**Table 5:** List of 10 conserved miRNAs which target disease resistance genes in potato leaves.

miRNA	Gene ID	Annotation
stu-miR482a-3p	PGSC0003DMG400020935	TMV resistance protein
stu-miR482b-3p	PGSC0003DMG402002428	TMV resistance protein
	PGSC0003DMG400013543	TMV resistance protein
	PGSC0003DMG400015693	TMV resistance protein
stu-miR482c	PGSC0003DMG402026432	Disease resistance protein
stu-miR482d-3p	PGSC0003DMG400011527	Disease resistance protein RPP13
	PGSC0003DMG400002980	Disease resistance protein RPP13
	PGSC0003DMG400006531	Disease resistance protein RPP13
	PGSC0003DMG400009455	Disease resistance RPP13
	PGSC0003DMG400024430	Disease resistance protein RPP13
	PGSC0003DMG400011527	Disease resistance protein RPP13
stu-miR482e-3p	PGSC0003DMG400027407	Disease resistance protein RPP13
	PGSC0003DMG400013090	TMV resistance protein
	PGSC0003DMG400029415	TMV resistance protein
	PGSC0003DMG400044837	TMV resistance protein
stu-miR6024-3p	PGSC0003DMG400019913	Disease resistance protein RPP13
stu-miR6025	PGSC0003DMG400018441	Putative late blight resistance protein
stu-miR6027	PGSC0003DMG400011898	Putative late blight resistance protein
	PGSC0003DMG400016600	Tospovirus resistance protein E
	PGSC0003DMG400006581	Putative late blight resistance protein
	PGSC0003DMG402032547	Disease resistance protein RPP13
stu-miR8038a-3p	PGSC0003DMG400009455	Disease resistance protein RPP13
stu-miR8038b-3p	PGSC0003DMG400009455	Disease resistance protein RPP13

**Figure 8:**
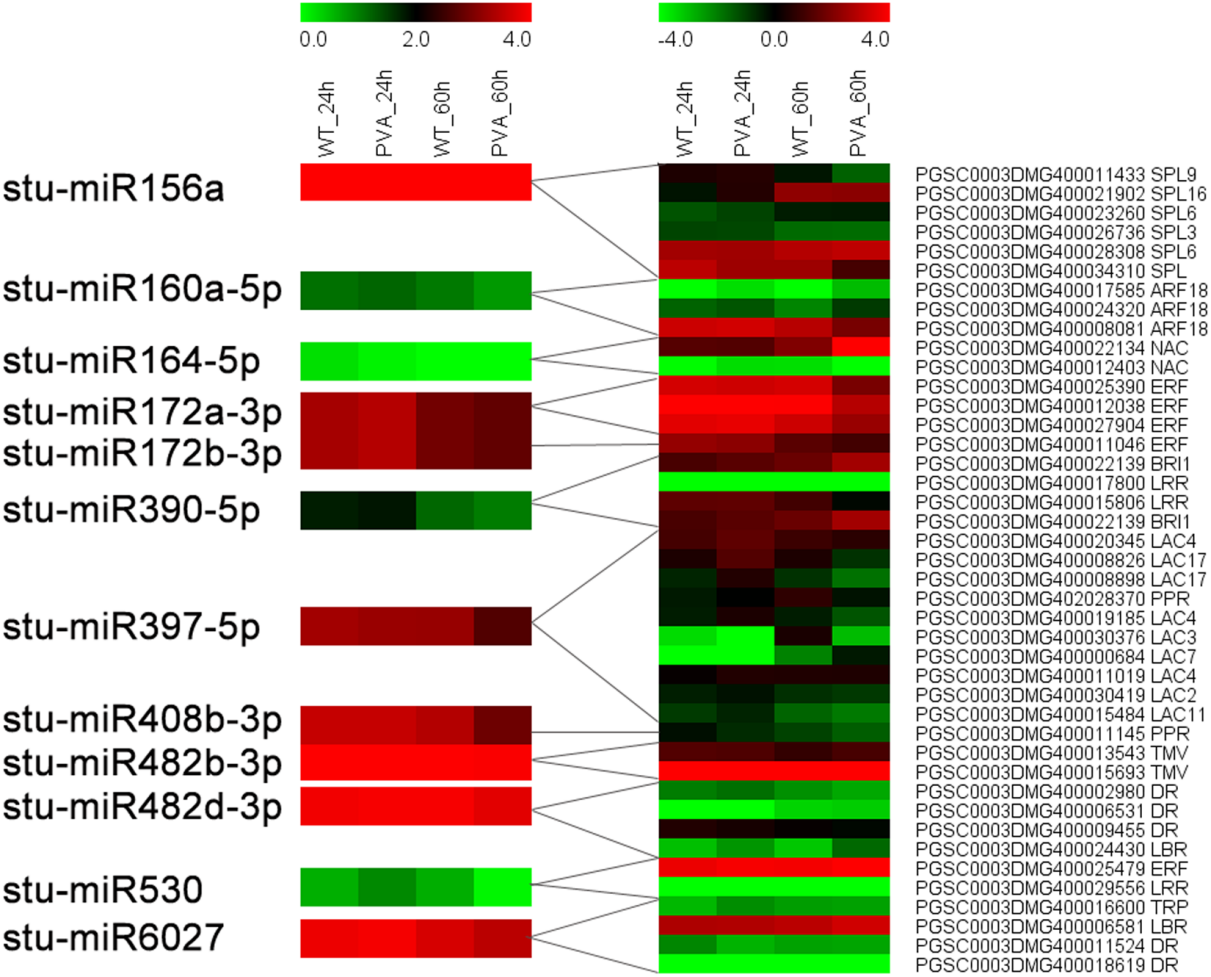
Heatmaps of differentially expressed miRNAs (DEMs) with their target genes.

A comparison of the expression level of miRNAs and mRNAs revealed miRNA-mRNA interactions during the PVA infection (Fig. 8 and Table S9). Many miRNAs were down-regulated with the expression levels of their targets increased at 60hpi (Fig. 8). Stu-miR164-5p which targeted an NAC TF (PGSC0003DMG400022134), was down-regulated at 24hpi and 60hpi. And the NAC target gene had a high expression level at 60hpi (Fig. 8). Furthermore, stu-miR390-5p and its target gene BRI1 (PGSC0003DMG400022139) which has been reported to be involved in the brassinosteroid signaling pathway, were shown to be negatively correlated on  the expression level (Fig. 8).

### Validation of the sequencing data by quantitative RT-PCR

**Figure 9:**
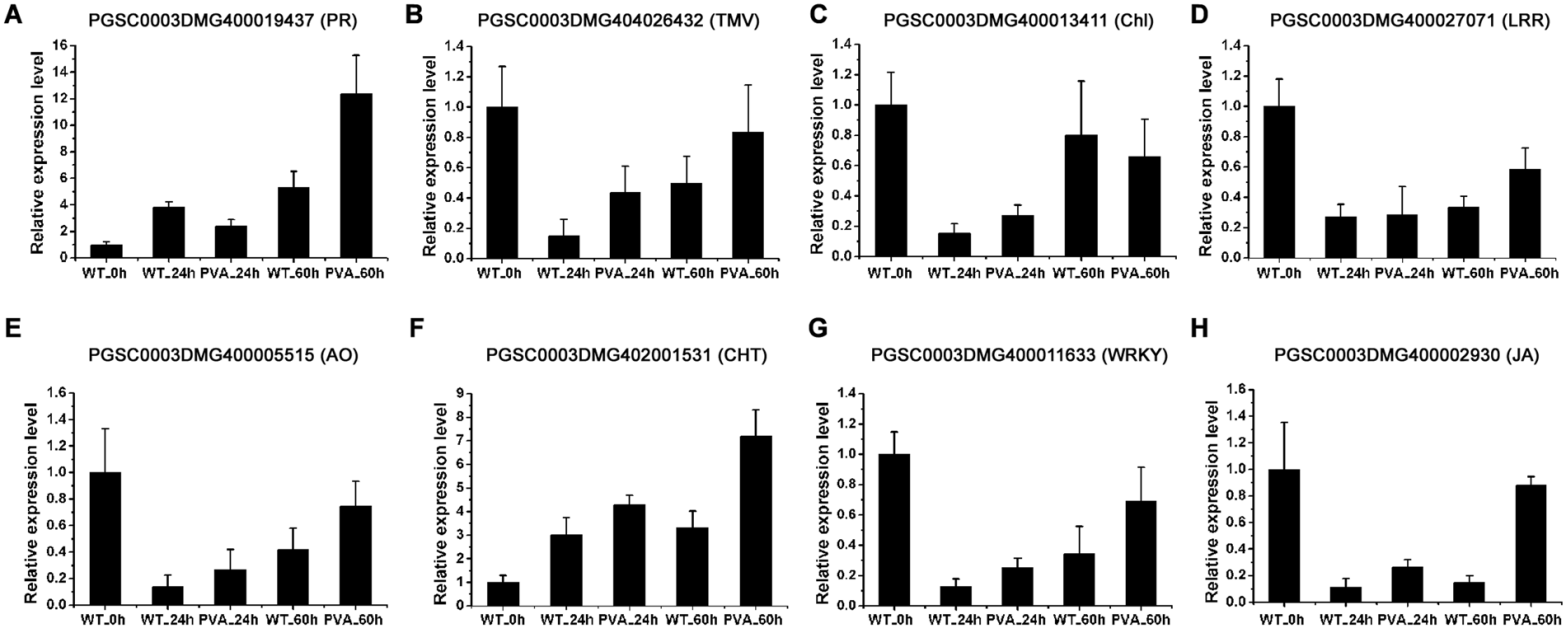
Quantitative RT-PCR (qRT-PCR) validation of differentially expressed genes (DEGs) at different time points (0h, 24 hpi and 60 hpi). PGSC0003DMG400019437 (PR, pathogenesis-related protein), PGSC0003DMG404026432 (TMV, TMV resistance protein), PGSC0003DMG400013411 (Chl, chlorophyll a-b binding protein), PGSC0003DMG400027071 (LRR, leucine-rich repeat receptor-like serine/threonine-protein kinase), PGSC0003DMG400005515 (AO, ascorbate oxidase), PGSC0003DMG 402001531 (CHT, chitinase), PGSC0003DMG 400011633 (WRKY, WRKY transcription factor), and PGSC0003DMG400002930 (JA, jasmonate ZIM-domain protein).

To validate the expression profiles of DEGs and DEMs from sequencing data, a qRT-PCR was performed. A total of 8 DEGs were randomly selected to validate the RNA-Seq expression profiles over the time course (Fig. 9). The PR protein (PGSC0003DMG400019437) and TMV resistance protein (PGSC0003DMG404026432) were highly expressed at 60hpi after inoculation, which was consistent with the RNA-seq data (Fig. 9A,B). And the expression level of one chlorophyll a/b binding protein (PGSC0003DMG400013411) was down-regulated at 60hpi (Fig. 9C). Leucine-rich repeat receptor-like protein kinase (PGSC0003DMG400027071) and ascorbate oxidase (PGSC0003DMG400005515) were also validated to have differential expression patterns in response to PVA infection (Fig. 9D,E). As a marker of the response to pathogens, chitinase (PGSC0003DMG402001531) was found to be up-regulated at 24hpi and 60hpi (Fig. 9F). In addition, WRKY TF (PGSC0003DMG400011633) and jasmonate ZIM-domain protein (PGSC0003DMG400002930) were also showed overexpressed at 60hpi after PVA inoculation (Fig. 9G,H). These qRT-PCR results were correlation with RNA-Seq data (correlation coefficient = 0.578).

**Figure 10:**
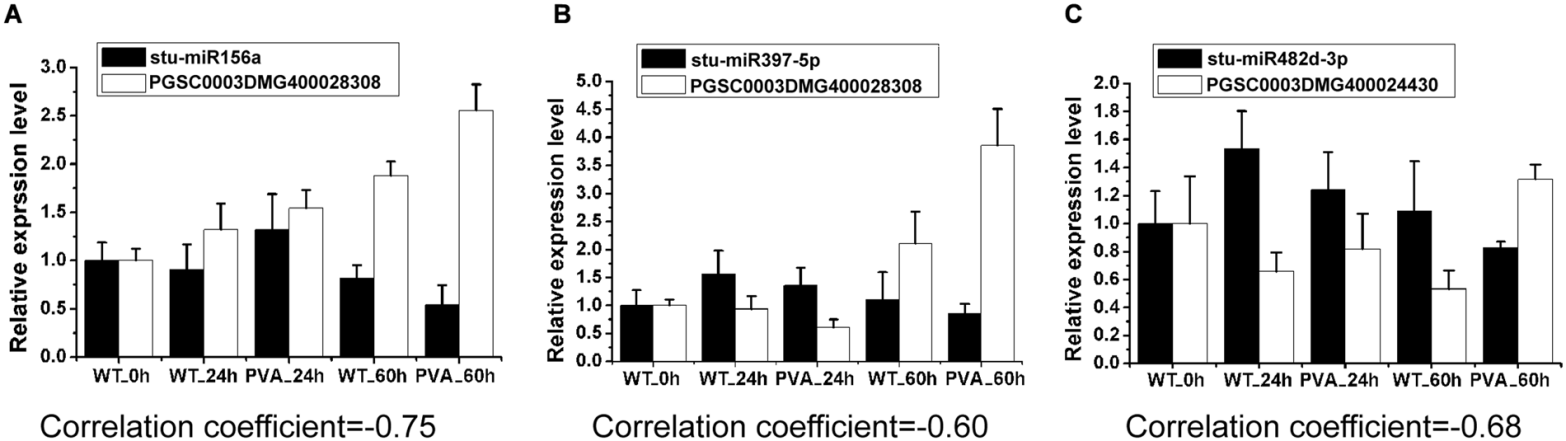
Quantitative RT-PCR (qRT-PCR) validation of three randomly selected differentially expressed miRNAs (DEMs) and their target genes at different time points (0 h, 24 hpi and 60 hpi). (**A**) stu-miR156a and its target PGSC0003DMG400028308 (SPL16). (**B**) stu-miR482d-3p and its target PGSC0003DMG400024430 (NB-ARC). (**C**) stu-miR397-5p and its target PGSC0003DMG400000684 (LAC7).

To validate the expression patterns of miRNAs and their targets, three known miRNAs and their corresponding target genes were randomly selected for qRT-PCR analysis (Fig. 10). The results showed that most of the miRNAs were negatively correlated with the expression level of their targets. In particularly, the expression level of the three miRNAs (stu-miR156a, stu-miR397-5p and stu-miR482-3p) were low at 60hpi, while their target genes were up-regulated (Fig. 10).

## Discussion

Potato virus A (PVA) is one of the most important viruses affecting potato production. To determine the molecular response to PVA and analyze the expression of disease resistance genes, we performed an integrated mRNA and miRNA profiling of potato leaves by high-throughput sequencing in this study. Our results provide new insights into the response to one of its most damaging pathogens in potato.

In this study, we investigated the transcriptome profiles of potato leaves in response to PVA infection using RNA-seq. GO and KEGG pathway analysis revealed that the stress response, defense response and plant-pathogen interaction pathways were enriched after PVA inoculation (Fig. 5 and Tables 3 and 4). A cascade of defense response genes were differentially expressed, leading to incompatible interactions between the host and pathogen (Fig. 3 and Table S4). These DEGs were involved in cell wall fortification, respiratory burst, kinase signaling, hormone signaling, transcription factors, and defense response genes (PR proteins, peroxidases, chitinases, laccases, lipoxygenase and phenyl ammonialyase). Although moderate correlation between Q-PCRs and RNA-seq for the different normalization standards and experimental methods, many DEGs were validated (Fig. 9). However, the activation of these defense response genes results in the synthesis of antimicrobial secondary metabolites, inhibiting the spread of PVA in potato.

Cell wall is the first physical barrier which protects plants from pathogens and prevents pathogens entering. In plants, the expression of cell wall related genes was regulated by biotic and abiotic stresses^32^. Peroxidase catalyzes active oxygen species (ROS) during the final step of cell wall fortification to polymerize lignin and the cross–linking of cell-wall components, stengthening the cell wall against the invading pathogen^33^. Our results showed that most of the peroxidase genes were up-regulated at 60hpi after PVA inoculation (Table S4). As the potential conductor of a symphony of signaling pathways, AO was apparently influenced under unfavorable environmental conditions^34,35^. Many of the AO genes were induced at the early stage of PVA inoculation (Fig. 3D). The expression level of L-ascorbate oxidase (PGSC0003DMG400005515) was validated by qRT-PCR, supporting the transcriptome data (Fig. 9E). The induction of chitinase can help to strengthen the cell wall by providing resistance^36^. Overexpression of chitinase in transgenic rice contributed to increased fungal resistance^37^. In the present study, a chitinase gene (PGSC0003DMG402001531) was significantly highly expressed at 60hpi (Fig. 9F and Table S4), indicating its role in resisting PVA infection.

PR genes are induced in plants in response to phytopathogens during the resistance processes. In this study, a total of 17 disease resistance genes were differentially expressed in potato leaves after PVA infection (Fig. 3A and Table S4). The defense response to fungus, defense response to bacterium and regulation of plant-type hypersensitive response were also overrepresented by GO analysis (Table 3). PR proteins are well known defense proteins in response to biotic or abiotic stresses in plants, especially, inducing by various types of pathogen^38^. PR4 proteins inhibited the growth of some pathogenic fungi and exhibited ribonuclease activity *in vitro* in wheat^39^. In this study, all the PR genes and most of the disease resistance or TMV resistance genes were highly expressed at 60 hpi comparing with the control at 60 h (WT60h). The expression level of PR (PGSC0003DMG400019437) and TMV (PGSC0003DMG404026432) was validated by qRT-PCR (Fig. 9A,B).

Virus infections often induce obvious symptoms in host plants, such as leaves with chlorosis, and these effects must reflect the perturbation of metabolism and signal pathways during plant development^40^. Pathogen attack always leads to changes in photosynthesis and photorespiration in the host plant, which has been shown to be involved in pathogen defense^41^. There were 56 photosynthesis-related genes differentially expressed after PVA inoculation (Fig. 3B and Table S4). Most of the photosynthesis-related genes were up-regulated at 24hpi, while they were down-regulated at 60hpi. The expression level of the chlorophyll a/b binding protein (PGSC0003DMG400013411) was validated the results. These results were consistent with previous studies on potato in response to PVY which showed that photosynthesis-related genes were up-regulated earlier than 4 hpi^11^. It was suggested that photosynthesis-related genes are important in incompatible reactions for elevating energy demand as the first response to stress^11,42^. The KEGG pathway also showed that “Oxidative phosphorylation”, “Starch and sucrose metabolism” and “Glycolysis/Gluconeogenesis” were enriched during the PVA infection (Tables 4 and S5).

In plants facing pathogen infection, hormonal signaling involving crosstalks between auxins, SA, JA, and ethylene are essential in the response to pathogens^43^. Consistent with previous results, GO enrichment analysis showed that the response to ABA, JA mediated signaling pathway and response to auxin were enriched in defense against PVA infection of potato (Figs 4,5A and Table 3). Jasmonate ZIM-domain protein 1 (PGSC0003DMG400002930) was significantly up-regulated at 60hpi (Figs 5B, 9 and Table S3). The two genes encoding salicylic acid-binding protein (PGSC0003DMG400025918 and PGSC0003DMG401025908) were also differentially expressed during PVA infection (Fig. 4 and Table S3). These results indicated that JA and SA signaling pathway were also involved in PVA defense (Fig. 6). As the receptors of pathogen signals, leucine-rich repeat (LRR) protein kinases were up-regulated in response to PVA infection (Figs 3C, 9D and Table S3). Mitogen-activated (MAP) kinases, calcium-dependent protein kinases and calcium-binding proteins also promote the signal transduction process (Fig. 5B and Table S4). Then, a transcriptional reprogramming occurred via activing PR genes, defense components and secondary metabolism among other processes^44^ (Fig. 6).

Transcription factors (TFs) are commonly reported to be involved in plant defense signaling pathways^30,45^. A total of 105 TFs belonging to 14 different families, were regulated during PVA infection, including WRKY, MYB, NAC, ARF and ERF (Tables S2 and S4). Most of the 16 WRKY TFs showed increased transcript abundance at both 24 and 60hpi (Fig. 4 and Table S4). The expression level of one WRKY (PGSC0003DMG400011633) was also validated by qRT-PCR (Fig. 9G). Previous studies reported that WRKY3, WRKY70, and WRKY75 are induced in other plant-pathogen interactions^46,47^. Thus, up-regulation of WRKY TFs indicated their likely roles in the regulation of transcriptional reprogramming associated with the early response to PVA in potato. Furthermore, ERF TFs were also induced in potato after PVA infection and they may participate in the activation of PR genes^48^. And the differentially expressed MYBs were involved in the regulation of disease resistance genes by regulating the expression of genes in phenylpropanoid and lignin biosynthesis^49^.

By regulating processes such as hormone balance, TFs and defense genes, miRNAs play vital roles in plant resistance to abiotic and biotic stresses^50^. In this study, we identified 113 conserved and 88 novel miRNAs which were differentially expressed in potato leaves (Table S7). Ten conserved miRNAs (stu-miR482a-3p and stu-miR482b-3p, etc) targeted the disease resistance genes, including TMV resistance protein, disease resistance protein and late blight resistance protein (Table 5). These targets all belong to the nucleotide binding site and leucine-rich repeat (NBS-LRR) class, which confers resistance to bacterial, fungal, or viral pathogens^51,52^. Previous study reported that overexpression of miR482e in potato resulted in hypersensitivity to *V*. *dahliae* infection^16^. In this study, we also found that stu-miR482d-3p which targets a disease resistance gene (PGSC0003DMG400024430) was down-regulated at 60hpi. The qRT-PCR results also supported the negative correlation of the expression level between stu-miR482d-3p and its target (Fig. 10C). These results suggested that the pathogenic miRNAs of potato were involved in PVA infection by targeting the disease resistance related genes.

In plants, miRNAs regulate gene expression mainly by cleaving the targeted mRNAs^13,53^. Many other miRNAs also participated in disease resistance by targeting defense genes or TFs. Stu-miR160a-5p targeted three ARF genes which are closely involved in auxin signal transduction (Fig. 8). Auxin signal transduction has been reported to be related to bacterial disease resistance in Arabidopsis^54^. Ten target genes of stu-miR397-5p encode laccase which is an important regulator in lignin metabolism^55^. And the expression of stu-miR397-5p was down-regulated at 60 hpi and validated by qRT-PCR (Fig. 10B and Table S7). The results indicated that stu-miR397-5p was invovled in the PVA infection. The target genes of stu-miR390-5p encoded LRR receptor-like serine/threonine-protein kinase (PGSC0003DMG400022139 and PGSC0003DMG400017800) which promote the signal transduction after PVA infection (Fig. 8 and Table S8). Stu-miR408a-3p which targets multicopper oxidase (PGSC0003DMG401027116 and PGSC0003DMG402023951) may scavenge the oxidase species during PVA infection^13^.

According to these results, we speculated that miRNAs could regulate the expression of certain genes and, through the changing expression of miRNAs, could play vital roles in regulating disease resistance in potato infected with PVA. The discovery of miRNAs involved in host defense responses to PVA will facilitate further studies of pathogen virulence and host resistance in potato. Further investigation of these miRNAs could identify their roles in the transcriptional regulation of defense response and plant-pathogen interactions.

## Conclusions

In this study, a global view of the transcriptional and post-transcriptional responses of potato for PVA infection was investigated by high-throughput sequencing. Our results showed that the induction of disease-related genes was highly expressed at 60 h after PVA inoculation. Our findings suggested that the differential regulation and expression of pathogenic miRNAs and disease-related genes played a central role in potato-PVA interactions. Further investigation will elucidate their functions whose expression changed the most after PVA infection. More functional analysis of the DEGs and DEMs would provide critical clues to reveal the molecular mechanism for PVA infection in potato. These genes or miRNAs may be useful for developing PVA resistant varieties in potato breeding.

**URLs:** AgriGO: http://systemsbiology.cau.edu.cn/agriGOv2/index.php; KEGG: http://www. genome. jp/kegg/. miRBase 20.0: http://www.mirbase.org.

## Electronic supplementary material

Supplementary Tables
